# Supplementary material for: ZmNAC17 Regulates Mesocotyl Elongation by Mediating Auxin and ROS Biosynthetic Pathways in Maize
Source: Int J Mol Sci. 2024 Apr 23;25(9):4585. doi: 10.3390/ijms25094585 (PMC11083593; doi:10.3390/ijms25094585)
Supplement: Supplementary file 1 [file ijms-25-04585-s001.zip › ijms-2928753-supplementary.pdf]

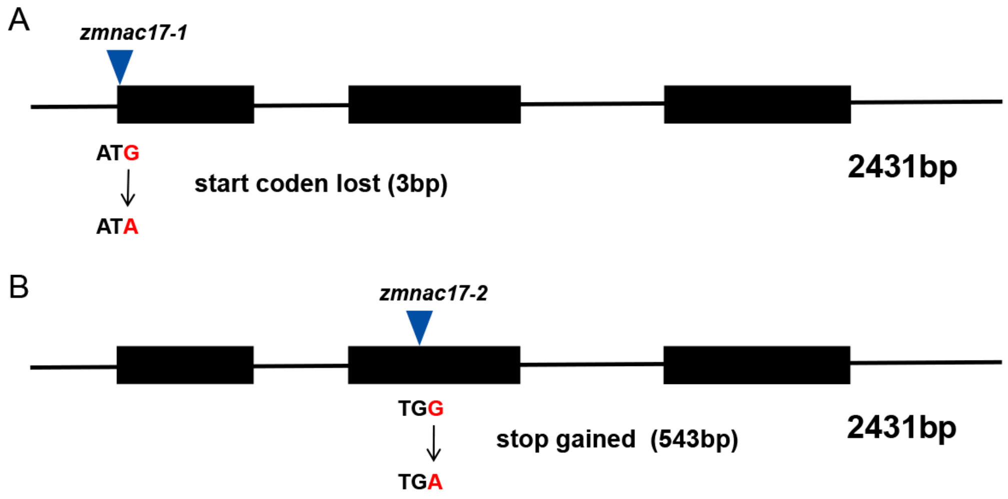

Figure S1. *zmnac17* mutation sites annotation. (A) *zmnac17-1* mutation site annotation. (B) *zmnac17-2* mutation site annotation.

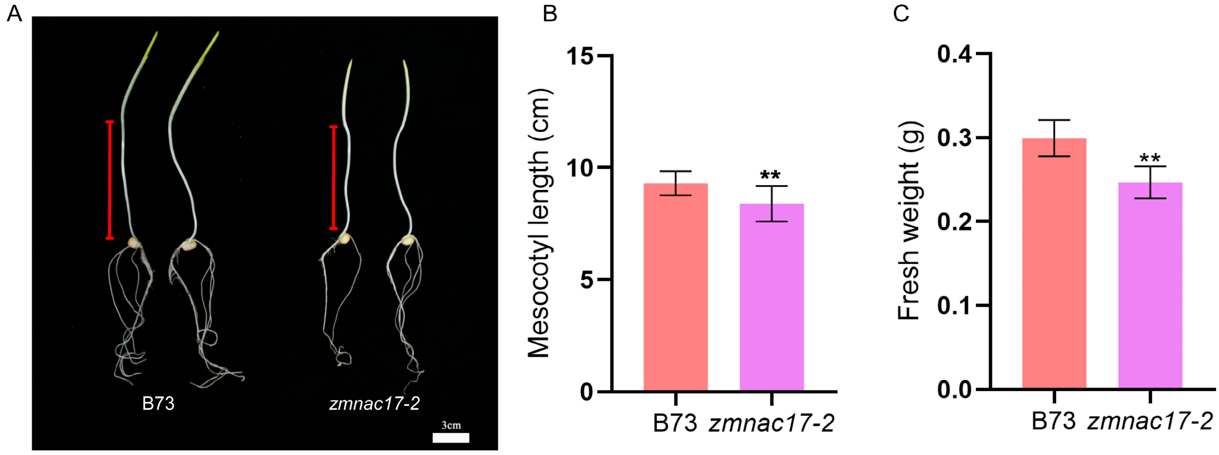

Figure S2. Phenotypic analyses of *zmnac17-2* mutant. (A) Seedling phenotypes of *zmnac17-2* grown in the dark for 7 days. Bars: 3 cm. (B) Results of quantification of mesocotyl length of B73 and *zmnac17-2* . (C) Results of quantification of mesocotyl fresh weight of B73 and *zmnac17-2*. Data are means ± SDs of at least 10 biological replicates. Statistical analysis conducted by using Student's unpaired t-test (** $p < 0.01$).
